# Resin Flow Behavior Simulation of Grooved Foam Sandwich Composites with the Vacuum Assisted Resin Infusion (VARI) Molding Process

**DOI:** 10.3390/ma5071285

**Published:** 2012-07-23

**Authors:** Chenhui Zhao, Guangcheng Zhang, Yibo Wu

**Affiliations:** 1 School of Natural and Applied Science, Northwestern Polytechnical University, Xi’an 710072, China; E-Mail: zhangguc@nwpu.edu.cn; 2 Luoyang Ship Material Research Institute, Luoyang 471023, China

**Keywords:** VARI, foam sandwich, foam grooves, resin flow behavior, simulation

## Abstract

The resin flow behavior in the vacuum assisted resin infusion molding process (VARI) of foam sandwich composites was studied by both visualization flow experiments and computer simulation. Both experimental and simulation results show that: the distribution medium (DM) leads to a shorter molding filling time in grooved foam sandwich composites via the VARI process, and the mold filling time is linearly reduced with the increase of the ratio of DM/Preform. Patterns of the resin sources have a significant influence on the resin filling time. The filling time of center source is shorter than that of edge pattern. Point pattern results in longer filling time than of linear source. Short edge/center patterns need a longer time to fill the mould compared with Long edge/center sources.

## 1. Introduction

Sandwich composites consist of two thin faces with high stiffness and high strength, and a core with low density and low stiffness. The faces are usually made of carbon fiber or glass fiber composites, and the core is typically made of end grain balsa wood or closed-cell polymer foams such as polyvinyl chloride (PVC) or polyurethane [1]. Foam sandwich composites have the advantages of high designability, light weight, high specific bending stiffness, strength, and good acoustical insulation *etc*. They are widely used in many applications such as buildings, aerospace systems, ship hulls and transportation [2,3]. However, their application is hindered by the high cost of the manufacturing process such as Resin Transfer Molding (RTM).

**Figure 1:**
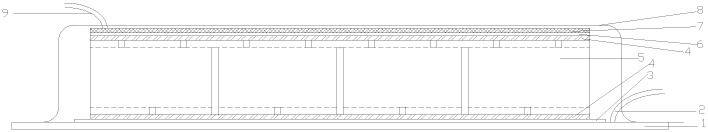
Experimental device schemes of the vacuum assisted resin infusion molding process (VARI).

VARI has been developed over the last decade and has clear advantages over the traditional RTM process since it eliminates the costs associated with matched metal tooling, reduces volatiles emission and allows the use of lower resin injection pressures [4,5,6,7]. The process enables the use of low-cost tooling while still producing high quality composite parts, making it the preferred manufacturing technique for current and future naval top-side structures (e.g., hulls, decks, and enclosures). The schematic of VARI is shown in Figure 1, which typically involves the following three steps. First, the preform is laid onto a rigid tool plate surface. The tool plate with the preform is surrounded by a formable vacuum bag. Second, the resin is injected through either a single or multiple inlet ports (depending on the preform size and shape) and transferred into the preform by a pressure gradient (induced by the vacuum pressure) and capillary effects. Third, curing of the impregnated preform is at the post-filling stage [8,9].

1—Glass mould; 2—Vacuum point source; 3—Release cloth; 4—Glass fabric; 5—Sandwich core; 6—Stripping cloth; 7—Diversion medium; 8—Vacuum bag; 9—Resin point source.

One of the most critical steps during the VARI process is the resin infiltration stage which ensures a complete infiltration of the preform (mold filling) with the resin. There are a lot of related works about fiber composites molded by the VARI process [10,11,12]. However, little research about foam sandwich composites via VARI technology have been reported.

The main objective of this paper is to study the flow behavior of the resin system via the VARI molding process to optimize technological parameters and provide fundamental knowledge for a practical production process. Since it costs time and money to study the resin flow behavior experimentally, a computer simulation method was applied to study flow behavior in the VARI process [13,14,15,16]. In this paper, the effect of high-permeable distribution medium (DM) and resin source pattern on resin flow behavior in the vacuum infusion molding process was studied through computer simulation and visualization experiments.

## 2. Results and Discussion

### 2.1. Influence of Ratio of DM/Preform on Resin Flow

DM is a porous material of high permeability, and is usually laid on the surface of preform to improve the resin flowing speed and shorten the resin filling time [17]. On the basis of grooves of the sandwich core providing a fast channel for resin flow, the effect of DM on resin flow behavior has been studied in this paper.

**Figure 2:**
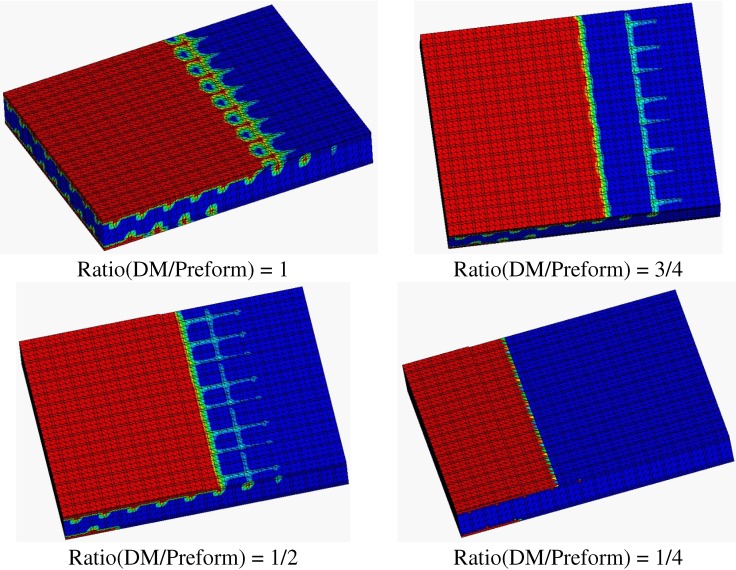
Sketch map of resin flow front at 90 s (distribution medium (DM)).

**Figure 3:**
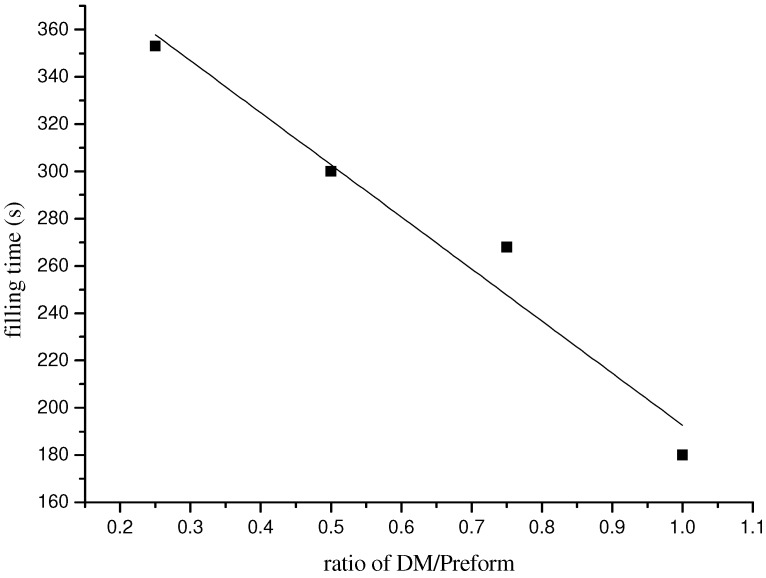
Relationship of filling time and ratio of DM/Preform.

The results of the influence of DM on the resin filling time are shown in Table 1 and Figure 2 and Figure 3. Table 1 indicates that the filling time simulated by PAM-RTM software is in good agreement with that of experimental results. In addition, distances of resin flow front obtained by simulation agree well with experimental observations. Both of these confirm the effectiveness of the simulation.

Simulation results in Table 1 show that DM can greatly reduce the mold filling time from 3768 s to 187 s in the vacuum infusion molding process. Figure 2 demonstrates that the resin flow over longer distance with a higher ratio of DM/perform shows the same time (90 s). The filling time is linearly reduced with the increase of the ratio of DM/Preform (Figure 3). In other words, flow rate rises with the increase of the ratio of DM area. These results agree with the findings by Yang [18], who studied the effect of DM on resin flow behavior in the VARI process using glass fiber reinforced materials as preform. The results also demonstrate that besides the fast channel for resin flow provided by grooves of the sandwich core, DM improves resin filling rate further.

**Table 1:** Relationship of ratio of diversion medium area and filling time.

Ratio of DM/preform	Filling time (s) (simulation results)	Filling time (s) (experiment results)
0	3768	3780
1/4	378	353
1/2	315	300
3/4	251	268
1	187	180

In addition, the flow pattern is modified by the introduction of DM. Without DM, only the grooves provide a fast channel for resin flow, and then the resin infiltrates into the woven glass fiber. In the presence of DM, both DM and grooves supply fast channels for resin flow, resulting in a shorter filling time.

### 2.2. Influence of Resin Source Pattern on Resin Flow

Resin source patterns can affect the resin flow pattern directly, and then influence the filling time. A common resin source form of the VARI process consists of point source, linear source, peripheral source, multilateral source and mixed source as seen in Table 2.

**Table 2:** Relationship of resin source pattern and resin flow pattern.

Souce pattern	Flow pattern
point source	radial flow
linear source	unidirectional flow
peripheral source	mixing flow
multilateral source	mixing flow
mixed source	mixing flow

**Figure 4:**
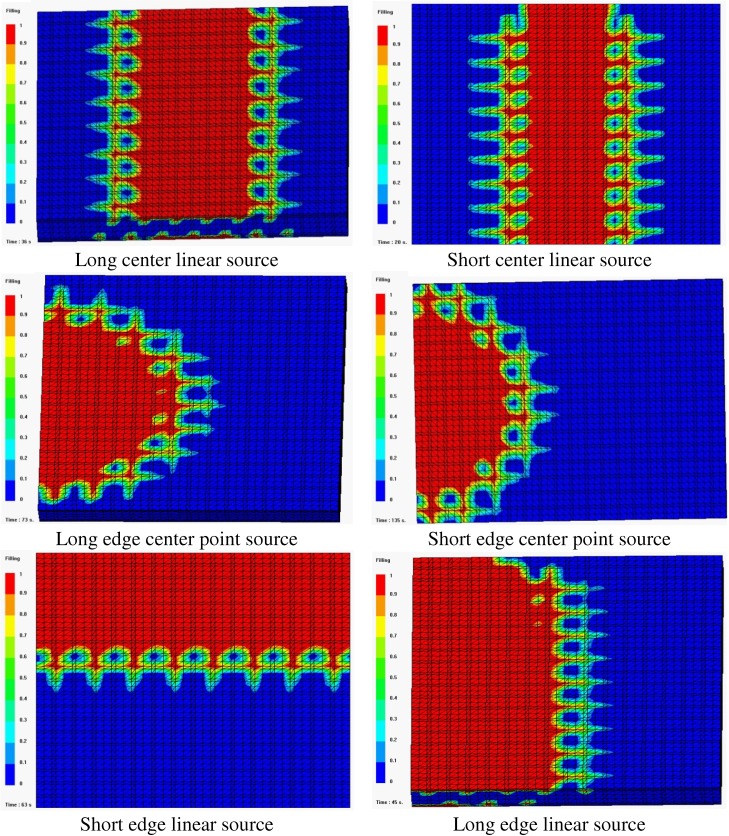
Sketch map of the resin flow front of different resin source patterns.

Although the resin source pattern of the VARI process varies, the basic pattern can be summed up as three types: linear source, point source and peripheral source. Limited to experimental conditions, this paper mainly compares point source and line source pattern. Experimental and simulation results are shown in Table 3 and Figure 4.

Table 3 shows that resin filling times with simulation results by PAM-RTM agree well with those of experimental observations, and resin flow patterns obtained from simulations (Figure 4) are in good agreement with experimental results. Moreover, the distance of the resin flow front from simulation is consistent with experimental observation.

**Table 3:** Influence of resin source pattern on filling time in the VARI process.

Resin source pattern	Filling time (s)	
	Simulation results	Experiment results
Short edge center point source	245	240
Long edge center point source	216	220
Short edge linear source	188	190
Long edge linear source	183	180
Short center linear source	100	105
Long center linear source	128	122

Figure 4 indicates that resin flow pattern is of unidirectional flow when using linear source, whereas resin flow pattern is of radial flow when using edge center point source. The filling time of linear source is shorter than that of point pattern (Table 3), which can be explained by the fact that in the same time more resin flows into the cavity, and the contact area of resin and medium is bigger. Short edge/center pattern results in a longer filling time than Long edge/center source, this may be caused by the difference of the flow distance (198 mm for Short edge/center pattern and 158 mm for Long edge/center pattern). Center source pattern needs a shorter time to fill the mould than edge pattern because the flow distance of the former is half of the latter. In a word, the resin source pattern has a big influence on resin flow pattern and filling time in grooved foam sandwich composites with the VARI process.

It also can be observed from both experiments and simulation that when edge center point source is adopted the resin flow pattern changes from radical flow to linear flow at about 200 s, which is different from the resin flow pattern in fiber preform without sandwich core [19]. This can be explained by the “fast track effect” of the grooves in the sandwich core. The resin fills the nearby grooves quickly and then these grooves replace the point source as the linear source, so that the resin flow mode changes to a linear flow later.

## 3. Experimental Section

### 3.1. Mathematical Model and Three-Dimensional Model (3D Model)

#### 3.1.1. Mathematical Model and Rationale

The resin flowing into a closed mold cavity can be represented as a flow through porous media. Darcy’s law is derived from general conservation equations by volume averaging techniques and is often used to model the resin flow in a porous medium. It enables the estimation of the average fluid velocity *v* (Darcy’s velocity) from the pressure gradient via ∇p the relationship [19,20,21,22,23,24,25,26,27,28]. Darcy’s law can be expressed as:

(1) $$v=-\frac{k}{\eta }\cdot \nabla p$$

where *η* is the viscosity of the fluid; K describes the permeability of the fibrous porous media. If the porous media is rigid (non-deformable), the continuity equation can be written as

(2) ∇⋅v=0

Equation (1) combined with the continuity Equation (2) will result in the following governing equation for the resin pressure:

(3) $$\nabla \cdot (\frac{k}{\eta }\cdot \nabla p)=0$$

Equation (3) is usually used to provide the pressure field for a given configuration. Flow velocity is then computed from Equation (1) to provide a description of the flow [19].

#### 3.1.2. Construction of 3D Model and Cell Division

**Figure 5:**
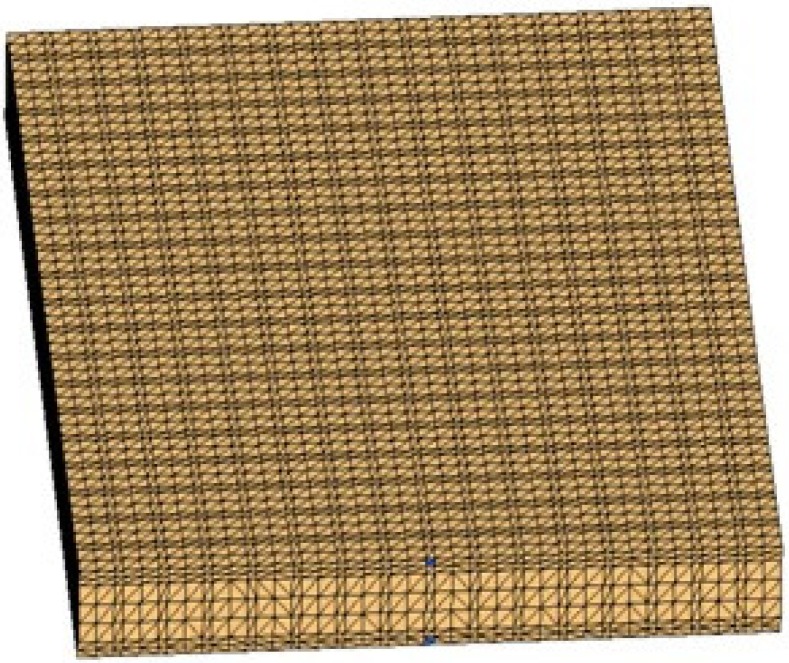
3D model of foam sandwich composite.

**Figure 6:**
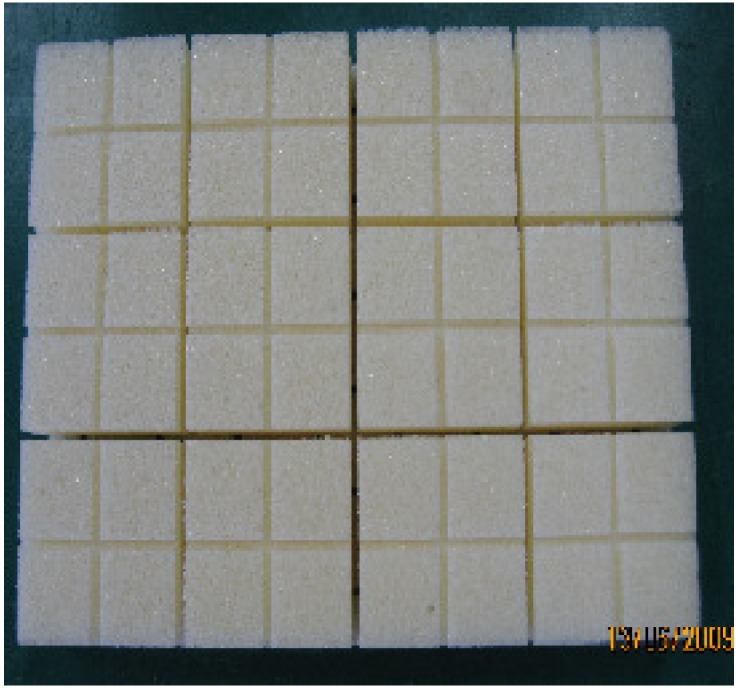
Physical model of foam sandwich composite.

A 3D model of a physical model is designed via UGNX6.0 and the triangle mesh unit divided by Visual-mash software. The size of the model (Figure 5) is 198 mm × 158 mm × 26.3 mm, and of the sandwich core 198 mm × 158 mm × 20 mm (Figure 6). A 3D model is imported into PAM-RTM. Permeability of glass fabric is 4.06 × 10^−10^ m^2^ (*k*_1_ = *k*_2_), and of DM is 6.12 × 10^−9^ m^2^ (*k*_1_ = *k*_2_). The thickness of glass fabric is 3 mm, and of DM is 0.3 mm. Porosity of glass is 0.47, and of DM is 0.85. The pressure of the mould is −1.01 × 105 Pa. The viscosity of the resin system is 270 mPa·s.

### 3.2. Materials and Experimental Design

#### 3.2.1. Materials

**Figure 7:**
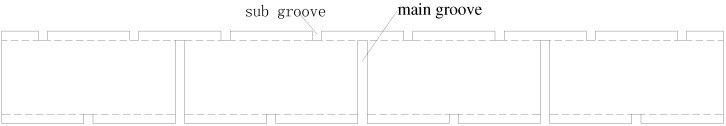
Front view of grooved polymethylacrylic imide (PMI) foam.

In the experiment bisphenol A, epoxy resin is chosen as the curing resin and amine as the curing agent, polymethylacrylic imide (PMI) is used as foam core. The length of PMI is 198 mm and the width 158 mm, with a height of 20 mm. The side of the main grooves of PMI is 2 × 18 mm for both width and height, and the pitch of the grooves is 40 mm, W × H of both the sub grooves on the surface and lower surface is 2 × 2 mm, Pitch of sub grooves on the surface is 20 mm, Pitch of sub grooves on the lower surface is 40 mm, as shown in Figure 7.

Auxiliary materials are glass fabric (reinforcing material), stripping film, diversion medium, vacuum bag, sealing adhesive tape, PTFE tube (liquid guiding pipe), and vacuum pump.

#### 3.2.2. Comparative tests of Ratio of DM/Preform

**Figure 8:**
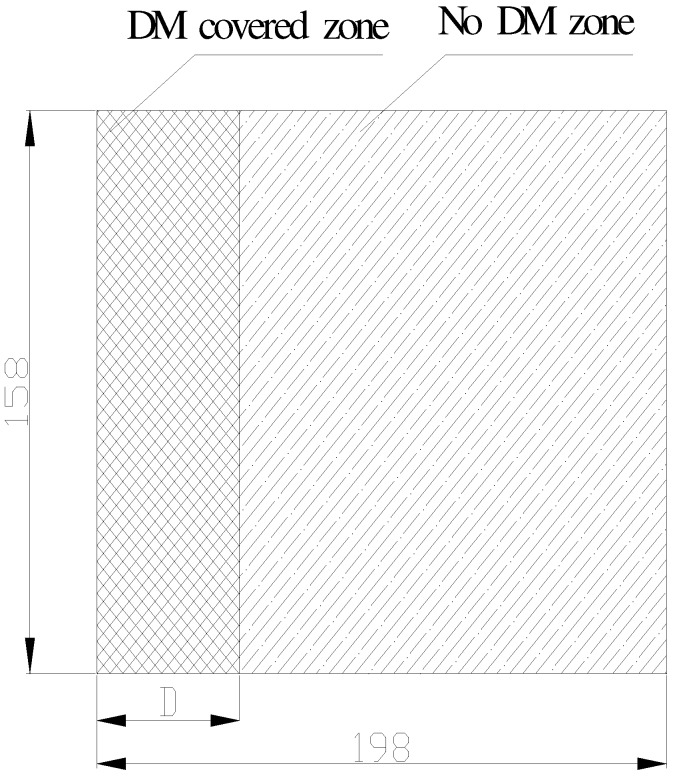
Short edge linear source.

**Figure 9:**
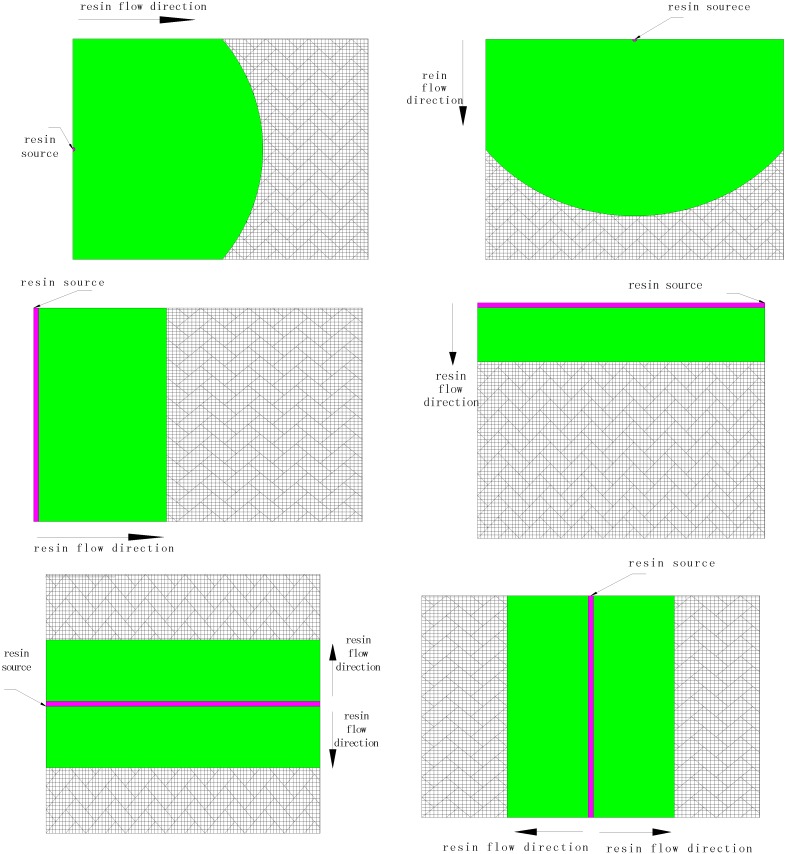
Schematic diagram of resin source pattern.

First, the relationship between the Ratio of DM/Preform and the resin flow behavior was investigated. DM was laid on the top surface of the preform (Figure 8). The ratio of the DM was changed by changing the length of the DM (D), since the width of the DM is the same as the preform. Different ratios of DM/perform were studied as in Table 4. Resin flow front and the corresponding time in the filling process were recorded from visualization flow experiments. The flow rate and the filling time were analyzed during the experiments. Short edge linear source was used as shown in Figure 9.

**Table 4:** Ratio of DM/preform.

Experiment number	DM/preform
1	0
2	1/4
3	1/2
4	3/4
5	1

#### 3.2.3. Comparative Tests of Resin Source Pattern

Six group contrast experiments were designed (Table 5 and Figure 9) to study the effect of resin source pattern on resin flow behavior. Flow time and flow front distance were recorded to evaluate the effect.

**Table 5:** Resin Source Pattern.

Experiment number	Resin source pattern
1	short edge center point source
2	long edge center point source
3	short edge linear source
4	long edge linear source
5	long edge center linear source
6	short edge center linear source

## 4. Conclusions

The flow behavior of the resin system via the VARI molding process has been studied experimentally and by computer simulation, the main conclusions can be drawn as follows:

DM is able to significantly reduce the molding filling time in grooved foam sandwich composites by the VARI process, and the mold filling time is linearly reduced with an increase of the ratio of DM/Preform. The existence of DM changes the resin flow model in the filling process.

The resin source pattern has a big influence on the resin flow pattern and filling time in grooved foam sandwich composites by the VARI process. Linear source leads to unidirectional flow while edge center point source results in radial flow. The filling time of linear source is shorter than that of point mode. Short edge/center mode results in a longer filling time than Long edge/center source. Center source pattern needs a shorter time to fill the mould than edge mode. The existence of the grooves in the sandwich core changes the resin flow mode by edge point source. Practically in production, linear source and center source can be chosen to shorten filling times.
